# Adult Willingness to Use Email and Social Media for Peer-to-Peer Cancer Screening Communication: Quantitative Interview Study

**DOI:** 10.2196/resprot.2886

**Published:** 2013-11-28

**Authors:** Sarah L Cutrona, Douglas W Roblin, Joann L Wagner, Bridget Gaglio, Andrew E Williams, Rosalie Torres Stone, Terry S Field, Kathleen M Mazor

**Affiliations:** ^1^University of Massachusetts Medical SchoolWorcester, MAUnited States; ^2^Meyers Primary Care Institutea joint endeavor of the University of Massachusetts Medical School, Fallon Community Health Plan and Reliant Medical GroupWorcester, MAUnited States; ^3^Center for Health ResearchKaiser PermanenteAtlanta, GAUnited States; ^4^Mid-Atlantic Permanente Research InstituteKaiser Permanente Mid-Atlantic StatesRockville, MDUnited States; ^5^Center for Health ResearchKaiser PermanenteHonolulu, HIUnited States; ^6^University of Massachusetts Medical SchoolDepartment of PsychiatryWorcester, MAUnited States

**Keywords:** colorectal neoplasms, electronic mail, social media, breast neoplasms, early detection of cancer, communication, health promotion, Internet, peer group, social support

## Abstract

**Background:**

Adults over age 40 are increasing their use of email and social media, raising interest in use of peer-to-peer Internet-based messaging to promote cancer screening.

**Objective:**

The objective of our study was to assess current practices and attitudes toward use of email and other e-communication for peer-to-peer dialogues on cancer screening.

**Methods:**

We conducted in-person interviews with 438 insured adults ages 42-73 in Georgia, Hawaii, and Massachusetts. Participants reported on use of email and other e-communication including social media to discuss with peers routine health topics including breast and colorectal cancer (CRC). We ascertained willingness to share personal CRC screening experiences via conversation, postcard, email, or other e-communication. Health literacy scores were measured.

**Results:**

Email had been used by one-third (33.8%, 148/438) to discuss routine health topics, by 14.6% (64/438) to discuss breast cancer screening, and by 12.6% (55/438) to discuss CRC screening. Other e-communication was used to discuss routine health topics (11.6%, 51/438), screening for breast cancer (3.9%, 17/438), and CRC (2.3%, 10/438). In the preceding week, 84.5% (370/438) of participants had used email, 55.9% (245/438) had used e-communication of some type; 44.3% (194/438) text, 32.9% (144/438) Facebook, 12.3% (54/438) instant message, 7.1% (31/438) video chat, and 4.8% (21/438) Twitter. Many participants were willing to share their CRC screening experiences via email (32.4%, 142/438 might be willing; 36.3%, 159/438 very willing) and via other e-communication (15.8%, 69/438 might be willing; 14.4%, 63/438 very willing). Individuals willing to send CRC screening emails scored significantly higher on tests of health literacy compared to those willing to send only postcards (*P*<.001).

**Conclusions:**

Many adults are willing to use email and e-communication to promote cancer screening to peers. Optimal approaches for encouraging peer-to-peer transmission of accurate and appropriate cancer screening messages must be studied.

## Introduction

### Electronic Peer Communication

The rise in use of email and social media among Americans over age 40 presents a unique opportunity for the development of novel health care interventions [1-6]. Electronic peer communication has been shown to influence political [7], consumer [8], and health-related behavior [9,10]. Internet-based peer-to-peer communication has the potential to act via a number of mechanisms, including information exchange, social support (eg, emotional and instrumental), and establishment of group norms [11]. Encouraging peer-to-peer promotion of healthy lifestyles and of cancer screening may be an effective way to further cancer prevention efforts in today’s rapidly changing and collaborative Web 2.0 environment [5,6,12,13].

### Web 2.0

Web 2.0 is a term used to describe the interactive experience of the Internet (in the form of blogs, wikis, Internet-based forums, etc.) [14], which has been made possible by technological advances that allow for and encourage open sharing of information. Increasingly, adults over age 50 share information using social media platforms that enable the interactive Web by engaging users who create content and communicate with their social network members (eg, Facebook, Twitter, and LinkedIn) [1,4,15]. Adults of all ages now go online to share their own experiences and to seek advice from friends and family on issues such as chronic disease caregiver roles and medical crises [12]. While medical illness may pose a more urgent prompt for peer-to-peer communication, a recent study indicates that several hundred breast cancer and colorectal cancer groups exist on Facebook and Twitter, and that cancer prevention is the main objective in over one-quarter of these groups [16]. We were interested in assessing the feasibility of a peer-to-peer intervention in which individuals who had completed cancer screening tests were invited to share their experiences with unscreened peers in order to promote completion of recommended screening behavior. We identified understanding current practices in Internet-based cancer screening discussion and gauging acceptability of such discussions as a necessary first step in developing our intervention.

In a diverse group of HMO-insured patients across three states (Georgia, Hawaii, and Massachusetts), we sought to document current practices and attitudes toward Internet-based email and social media cancer screening discussions. We also explored willingness to use these avenues for future peer communication and the association between health literacy and likely mode of communication.

## Methods

### Study Population and Setting

This study was conducted within the Cancer Research Network (CRN), a consortium of research organizations affiliated with 14 community-based nonprofit integrated health care delivery systems and the National Cancer Institute. Participants were recruited from three health plans–Kaiser Permanente Georgia (KPGA), Kaiser Permanente Hawaii (KPHI), and Fallon Community Health Plan (FCHP). This study was reviewed and approved by the Institutional Review Boards at each of the plans.

Participants in the present study had previously completed a two-hour study session for a larger study focused on communication of cancer information [17]. One CRN site—Kaiser Permanente Colorado—participated in the previous larger study, but not in the present study. All participants were 40-70 years of age at the time of recruitment for the larger study (some were 71 by the time the interviews occurred), all had been a member of one of the participating health plans for a minimum of 5 years, were able to understand English, and had no physical or mental limitation that would preclude participating in a two-hour in-person interview. We targeted this age range because these adults are most likely to face cancer screening decisions and to be at elevated risk for most cancers compared to younger adults. To optimize sampling across educational levels, at FCHP, KPGA and KPHI, sampling was stratified by United States Census-based estimates of educational level defined by the percentage of residents with a high school education or less in the census tract in which participants lived. At KPGA, sampling was further stratified according to the percent of African-American residents, to ensure that African-American and white members were invited in equal numbers within each educational strata. A variety of recruitment techniques were used, including mailings, telephone follow-up, and offering sessions at multiple locations. Interested participants were screened to confirm ability to communicate in English, adequate corrected hearing and vision, and the absence of physical or psychological limitations that would preclude participation. Study sessions lasted approximately 2 hours, and were conducted in-person by a trained research assistant. All items (except reading items) were administered orally. A total of 1074 participants completed interviews between June 22, 2009 and April 19, 2010.

For the present study, 3 sites participated (KPGA, KPHI, and FCHP). There were 789 participants from the initial study that were contacted by mail; approximately one week later, individuals who did not respond were contacted via telephone to again extend the invitation to participate. There were 438 (56% of the 789 people invited) people who agreed to participate. For budgetary reasons, participants from FCHP were recruited more aggressively and made up a higher proportion of this current study population (46.3%, 203/438 of the present study sample was from FCHP as compared to 28.86%, 310/1074 of the previous larger study). This higher proportion of FCHP participants resulted in a higher proportion of white participants. There were no significant differences in age, educational level, health literacy scores, numeracy scores, or self-reported health status for current study participants from the 3 sites as compared to previous participants at these 3 sites.

Interviews were conducted for the present study between August 4, 2011 and January 27, 2012. Sessions lasted approximately 1 to 1.5 hours (see Multimedia Appendix 1).

### Data Collection

Health literacy assessments were conducted during the previous study’s sessions. Comprehension of spoken health messages (sometimes referred to as verbal health literacy) was assessed using the Cancer Message Literacy Test-Listening (CMLT-Listening). This test is administered via computer and requires no reading. Development of this test is described in further detail elsewhere [18]; results of reliability and validity studies are described by Mazor et al [17]. Print literacy was assessed using the Cancer Message Literacy Test-Reading (CMLT-Reading) [17,18]. Numeracy was assessed using the Lipkus numeracy scale [19]. Self-efficacy was assessed using the Perceived Efficacy in Patient-Physician Interactions (PEPPI) [20]. Aside from the CMLT-Reading, research staff administered the measures verbally.

During in-person interviews, 438 returning participants engaged in the current study reported on their recent use of email and other electronic communication. E-communication included texting, Facebook, instant messaging, Internet-based and video chatting, Twitter, and LinkedIn. We queried participants on their use of these media: (1) for any purpose, (2) to discuss routine health-related topics (including cancer screening, vaccines, diet, or exercise), and (3) for specific types of cancer screening; colorectal cancer (CRC) and breast cancer. Participants were also questioned regarding their willingness to communicate about such topics using email and other forms of e-communication.

In order to explore the role of user-generated content, participants were provided with the following hypothetical situation–“Imagine that you completed colon cancer screening. Everything went OK and your results were fine. The doctor asked you to help educate friends and family members over age 50 about colon cancer screening. We are trying to design a message to be sent out by people who have completed colonoscopies, so that they can explain to friends and family why screening is important. Please help us design a message you’d be willing to pass along to friends and family members over age 50.”

**Figure 1:**
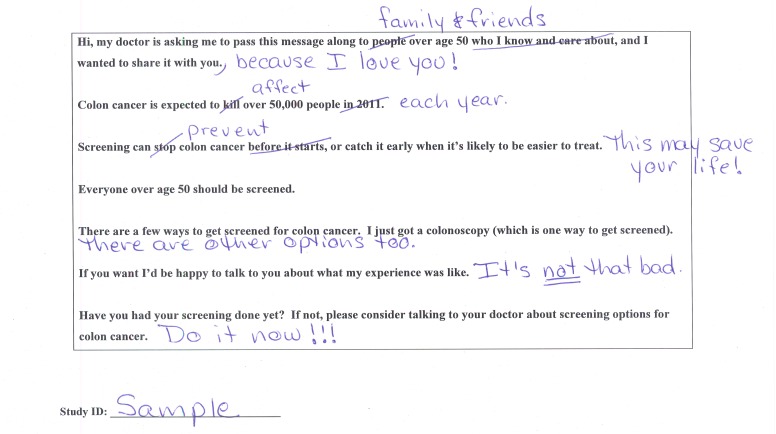
Colorectal cancer screening message template with edits (example).

We then provided participants with a sample message in which the sender shares the fact that he or she has completed a colonoscopy and urges readers to discuss CRC screening with their doctor (Figure 1 shows the sample message). We encouraged participants to edit the message as they wished, then asked whether they would be willing to send the edited message to friends and family by either email or postcard. No messages were actually sent. Those who indicated they would not be willing to pass along their message (“nonsenders”) were asked to explain why and their answers were transcribed and categorized.

**Figure 2:**
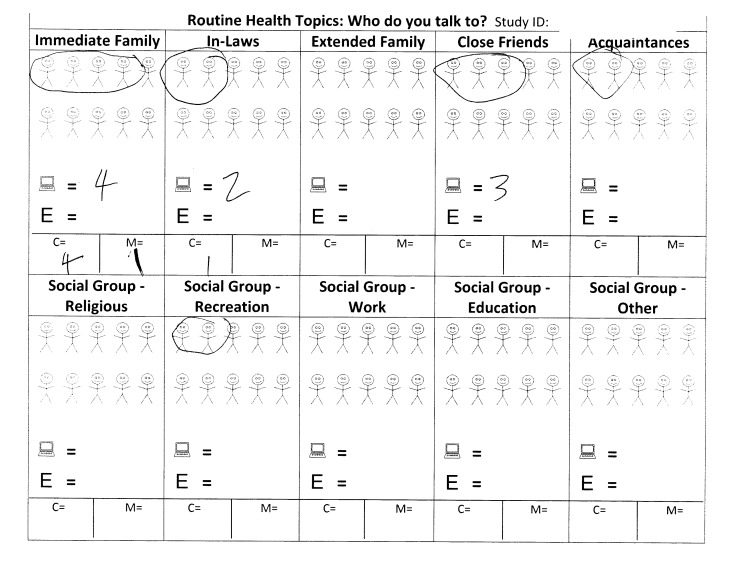
Health communication network tool. Study participants were provided with blank worksheets and asked: “Please mark one stick figure for each person you can think of that you communicate with about routine health topics…these are people you would communicate with about routine health topics like cancer screening, vaccine shots, diet, or exercise.” The worksheet was used to facilitate the estimation of the number of emails or postcards promoting colorectal cancer screening that they would send to members of their social network.

Participants who indicated that they would be willing to pass along messages were asked to estimate the number of emails or postcards they would send. To facilitate this estimation, participants were offered a worksheet (Figure 2 shows this worksheet) and encouraged to circle stick figures in each of 10 social group categories in order to visually designate members of their social network with whom they communicate about routine health topics and cancer screening.

### Analysis

We calculated the number of people who reported using email and e-communication for: (1) any use, and (2) discussion of routine health topics including cancer screening. We used χ^2^ to analyze bivariate associations between age group and use of email or e-communication. Then, focusing on CRC (since this screening is applicable to both men and women), we analyzed willingness to share CRC screening experience via various modes (through general conversation, email, or other e-communication; or through a specific self-edited message via email or postcard). For this analysis we again used χ^2^ to analyze bivariate associations between age and willingness to share via various modes. Finally, we sought to understand whether sociodemographic factors or measures of health literacy, numeracy, or self-efficacy were associated with willingness to share CRC screening experience via email or postcard. We conducted a multinomial logistic regression model (generalized logistic regression) using SAS 9.2 (SAS Institute, Inc, Cary, NC), modeling the odds of being: (1) an email sender, or (2) a postcard sender, as compared to (3) being a nonsender. We then conducted a logistic regression modeling the odds of being a sender of either email or postcard. We included in the model variables identified a priori as being of interest.

## Results

### Study Participants

The majority of our study participants (52.3%, 229/438) were 60 years or older and there were slightly more women (56.4%, 247/438) than men (See Table 1). There were three-quarters (75.6%, 331/438) reporting educational levels above a high school degree. Almost 90% (382/438) of all participants reported ever having completed any type of CRC screening and 72.6% (318/438) reported having had a colonoscopy.

**Table 1:** Participant characteristics. χ^2^ used to derive *P* values shown for age, gender, race/ethnicity, education, marital status, ever had friends/family diagnosed with CRC, and ever had a colonoscopy. Analysis of variance–ANOVA used to derive *P* values for health literacy scores, numeracy, and self-efficacy.

Characteristic		n	%
Study sample		438	100.0
**Study site**			
	Georgia	130	29.7
	Hawaii	105	24.0
	Massachusetts	203	46.3
**Race/ethnicity**			
	Black/African-American	65	14.8
	Asian/Pacific Islander	45	10.3
	White/Caucasian	286	65.3
	Other or not reported	42	9.6
**Language spoken at home**			
	English	419	95.7
	English and other	9	2.1
	Other	7	1.6
**Education**			
	High School degree or less (includes technical school)	104	23.7
	At least some college	331	75.6
**Age (in years)**			
	40-49	52	11.9
	50-59	157	35.8
	60-73	229	52.3
**Gender**			
	Male	191	43.6
	Female	247	56.4
**Marital status**			
	Married	282	64.4
	Unmarried	153	34.9
**Work status**			
	Working for pay	260	59.4
	Retired	126	28.8
	Disabled	17	3.9
	Other	35	8.0
**Self-reported health status**			
	Excellent/very good	240	54.8
	Good/fair/poor	197	45.0
**Number of comorbidities**			
	0/1	336	76.7
	2+	99	22.6
**Current smoking status**			
	Current smoker	27	6.2
	Current nonsmoker	410	93.6
**Has doctor ever recommended that you be screened for CRC cancer?**			
	Yes	385	87.9
	No	48	11.0
**Completed any type of CRC screening?**			
	Yes	382	87.2
	No	50	11.4
**Ever had a colonoscopy?**			
	Yes	318	72.6
	No	114	26.0
**Health literacy, numeracy, and efficacy measures, mean (SD)**			
	Verbal health literacy (CMLT-Listening)	79.9	(14.1)
	Print health literacy (CMLT-Reading)	84.8	(14.6)
	Numeracy	78.5	(21.9)
	Self-efficacy (PEPPI)	8.1	(1.3)

### Use of Email for Discussions of Routine Health Topics and Cancer Screening

A high percentage of participants (84.5%, 370/438) had used email in the past week with no significant variation across age categories (Table 2). Only one-third of the participants (33.8%, 148/438) had ever used email to discuss routine health topics, and more than one in ten had used email to discuss CRC screening (12.6%, 55/438) or breast cancer screening (14.6%, 64/438). There was no significant variation by age category for these measures.

### Use of Electronic Communication for Discussions of Routine Health Topics and Cancer Screening

In the previous week, just over half of all participants (55.9%, 245/438) had used some other form of electronic communication (including texting, Facebook, instant messaging, Internet-based or video chatting, Twitter, LinkedIn, or other), there was significant variation by age with the youngest age group (40-49 year olds) being most likely to report use (Table 2). Approximately one in ten respondents (11.6%, 51/438) had ever used electronic communication (other than email) to discuss routine health topics, as expected from trends in overall use, youngest respondents were most likely to report such behavior. Similarly, close to one in ten participants under age 60 (8.6%, 18/209) had used electronic communication to discuss CRC screening or breast cancer screening. Texting and Facebook were the two most commonly mentioned forms of electronic communication across all age categories both for general use and specifically for discussion of routine health topics.

**Table 2:** Use of email and social media to discuss health-related topics and to discuss cancer screening.

		Total sample (N=438)		Age 40-49		Age 50-59		Age 60-73		*P*
		n	%	n	%	n	%	n	%	
**Used email**										
	Ever	387	88.4	49	94.2	144	91.7	194	85.5	.067
	In past week	370	84.5	47	90.4	135	86.0	188	82.8	.345
	For 5-7 days in past week	303	69.2	39	75.0	120	76.4	144	63.4	.016
	To discuss routine health topics	148	33.8	14	26.9	62	39.5	72	31.4	.180
	To discuss CRC screening^a^	55	12.6	6	11.5	24	15.3	25	10.9	.683
	To discuss breast cancer screening^a^	64	14.6	9	17.3	26	16.6	29	12.7	.176
**Used other e-communication** ^b^										
	Ever	247	56.4	43	82.7	107	68.2	97	42.4	<.001
	In past week	245	55.9	43	82.7	105	67.7	97	42.4	<.001
	For 5-7 days in past week	148	33.8	30	57.7	70	45.2	48	21.0	<.001
**Type of e-communication used**										
	Texting	194	44.3	41	78.8	88	56.1	65	28.4	<.001
	Facebook	144	32.9	24	46.2	59	37.6	61	26.6	.008
	Instant messaging/Internet-based chatting	54	12.3	10	19.2	28	17.8	16	7.0	.002
	Video chatting	31	7.1	5	9.6	16	10.2	10	4.4	.068
	Twitter	21	4.8	4	7.7	13	8.3	4	1.7	.007
	LinkedIn	3	0.6	0	0.0	3	1.9	0	0.0	.067
	Other	3	0.7	1	1.9	1	0.6	1	0.4	.500
Used other e-communication to discuss routine health topics		51	11.6	16	30.8	25	15.9	10	4.4	.000
Used other e-communication to discuss CRC screening^c^		10	2.3	3	5.8	7	4.5	0	0.0	.168
Used other e-communication to discuss breast cancer screening^c^		17	3.9	7	13.5	9	5.7	1	0.4	.244
**Type of e-communication used to discuss routine health topics**										
	Texting	31	7.1	11	21.2	16	10.2	4	1.7	.000
	Facebook	21	4.8	5	9.6	10	6.4	6	2.6	.053
	Instant messaging/ Internet-based chatting	6	1.4	3	5.8	2	1.3	1	0.4	.011
	Video chatting	3	0.7	0	0.0	2	1.3	1	0.4	.505
	Twitter	1	0.2	0	0.0	1	0.6	0	0.0	.408
	Other	2	0.5	1	1.9	1	0.6	0	0.0	.163
Uses cell phone to access Internet		122	27.9	23	44.2	55	35.3	44	19.2	.000

^a^Only asked of those who use email to discuss routine health topics.

^b^Includes texting, Facebook, instant messaging, Internet-based or video chatting, Twitter, LinkedIn or other.

^c^Only asked of those who use e-communication to discuss routine health topics.

### Attitudes Toward Discussing CRC Screening via Email and Electronic Communication

When asked whether they would be willing to share their CRC screening experience with friends or family in order to educate and encourage screening, close to three-quarters of all participants (73.3%, 321/438) were “very willing” to share through conversation, with almost all of the remaining stating that they “might be willing” to share in this way (Table 3). Email and other electronic communication showed lower proportions of users who were “very willing” to share (41.3%, 159/385 email users; and 25.1%, 63/251 e-communication users), but over half of both user groups would at least consider sharing their CRC experience in this way (“might be willing” or “very willing” to share–78.2%, 301/385 of email users; and 52.6%, 132/251 of e-communication users).

When offered the opportunity to create their own content, adding, deleting, or rearranging text according to their own preferences (Figure 1), the vast majority of participants (85.4%, 374/438) were willing to send a message encouraging CRC screening; 68.5% (300/438) indicated that they would use either email or a combination of email and postcard (Table 3). Across all age groups, those who would send emails were in the majority and those who would send only postcards were the next largest group (Table 4). Older participants were least likely to send any message and men were more likely than women to indicate that they would not send. Those with a higher educational level were more likely to choose email, but education was not associated with overall willingness to send (Table 4).

**Table 3:** Willingness to share personal CRC screening experience with friends and family and preferred mode.

Mode by which CRC screening experience would be shared		Total sample		Age 40-49		Age 50-59			Age 60-73			*P*
		n=433	%	n=52	%	n=156		%	n=225		%	
**Through conversation asked of everyone (n=433)**												.620
	Not willing	10	2.3	0	0.0	4		2.6	6		2.7	
	Might be willing	102	23.6	13	25.0	32		20.5	57		25.3	
	Very willing	321	74.1	39	75.0	120		76.9	162		72.0	
**By email asked only of those who use email (n=385)**												.393
	Not willing	84	21.8	8	16.3	30	21.1		46	23.7		
	Might be willing	142	36.9	18	36.7	47	33.1		77	39.7		
	Very willing	159	41.3	23	46.9	65	45.8		71	36.6		
**By other electronic communication asked only of those who use e-communication (n=251)**												.029
	Not willing	119	47.4	13	30.2	48	45.3		58	56.9		
	Might be willing	69	27.5	18	41.9	27	25.5		24	23.5		
	Very willing	63	25.1	12	27.9	31	29.2		20	19.6		

**Table 4:** Characteristics of respondents who are willing to pass along self-edited email or postcard messages sharing CRC screening experience (n=432).

						Email^a^			Postcard only				Would not send			*P*
						n=300		%	n=74		%		n=58	%				
**Age, n %**																.010
			40-49			42		82.4	5		9.8		4	7.8		
			50-59			118		76.1	20		12.9		17	11.0		
			60-73			140		61.9	49		21.7		37	16.4		
**Gender, n %**																.010
			Male			117		62.2	37		19.7		34	18.1		
			Female			183		75.0	37		15.2		24	9.8		
**Race/ethnicity, n %**																.365
			Black/African-American			46		70.8	15		23.1		4	6.2		
			Asian/Pacific Islander			35		77.8	5		11.1		5	11.1		
			White/Caucasian			192		68.3	47		16.7		42	14.9		
			Other or not reported			27		65.9	7		17.1		7	17.1		
**Education, n %**																<.001
			High School degree or less (includes technical school)			55		53.9	33		32.4		14	13.7		
			At least some college			243		74.3	41		12.5		43	13.1		
**Marital status, n %**																.561
			Married			198		71.2	45		16.2		35	12.6		
			Unmarried			100		66.2	29		19.2		22	14.6		
**Ever had friends/family diagnosed with CRC?, n %**																.063
			Yes			103		76.9	19		14.2		12	9.0		
			No			196		66.0	55		18.5		46	15.5		
**Ever had a colonoscopy?, n %**																.039
			Yes			221		70.4	58		18.5		35	11.1		
			No			73		65.2	16		14.3		23	20.5		
Verbal health literacy “CMLT-Listening”, mean (SD)						81.24 (13.50)			73.96 (15.11)				81.26 (13.46)			<.001
Print health literacy “CMLT-Reading”, mean (SD)						86.23 (13.72)			78.51 (15.60)				85.39 (15.85)			<.001
Numeracy, mean (SD)						79.72 (20.18)			71.62 (27.63)				80.82 (21.99)			.012
Self-efficacy “PEPPI”, mean (SD)						8.18 (1.21)			8.22 (1.51)				7.56 (1.67)			.004

^a^Those indicated in the email column would send out either only emails or would send a mix of emails and postcards.

### CRC Screening Email Messages: Role of Health Literacy and Self-Efficacy

Using ANOVA tests, mean measures of health literacy (print and verbal) and numeracy were compared across email senders, postcard senders, and those who wouldn’t send (Table 4). We found a consistent pattern across these three categories, with postcard senders scoring significantly lower than the other 2 groups on all three measures. For measures of self-efficacy, senders (both email and postcard) scored significantly higher than those who wouldn’t send.

On multivariate analysis (Table 5, Model 1), those with lower education were significantly more likely to be postcard senders than to be nonsenders. When we modeled the odds of sending any message at all (Table 5, Model 2), neither education nor health literacy level was significant. Those with higher self-efficacy scores were more likely to send messages in both models.

**Table 5:** Willingness to send messages sharing CRC screening experience with peers.

		Model 1^a^				Model 2^b^	
		Odds of being an email sender		Odds of being a postcard sender		Odds of sending either email or postcard	
		OR	95% CI	OR	95% CI	OR	95% CI
**Age**							
	40-49	2.21	0.73-6.73	0.77	0.19-3.22	1.83	0.61-5.53
	50-59	1.72	0.89-3.29	1.06	0.47-2.41	1.57	0.83-2.97
	60 and up^c^	-	-	-	-	-	-
**Gender**							
	Male	0.50	0.27-0.90	0.80	0.38-1.67	0.55	0.31-0.99
	Female^c^	**-**	**-**	-	-	**-**	**-**
**Race/ethnicity**							
	Black/African American	2.55	0.70-9.26	2.70	0.66-11.07	2.50	0.70-8.92
	Asian/Pacific Islander	1.19	0.46-3.12	0.59	0.15-2.32	1.05	0.41-2.72
	Other or not reported	0.85	0.31-2.30	0.93	0.28-3.14	0.84	0.32-2.20
	White/Caucasian^c^	-	-	-	-	-	-
**Education**							
	High School degree or less (includes technical school)	0.88	0.42-1.88	2.39	1.02-5.59	1.17	0.56-2.45
	At least some college^c^	-	-	**-**	**-**	-	-
Print health literacy score^d^ (CMLT-Reading)		1.01	0.99-1.03	0.99	0.96-1.01	1.00	0.98-1.03
Self-efficacy (PEPPI)^d^		1.25	1.03-1.53	1.35	1.04-1.74	1.28	1.06-1.55

^a^Model 1–Odds of being an email sender or a postcard sender as compared to being a nonsender.

^b^Model 2–Odds of being a sender, either email or postcard, as compared to being a nonsender.

^c^reference

^d^per unit increase in score

### CRC Screening Email Messages: How Many Would Be Sent?

Those who indicated they would be willing to send emails estimated that they would send, on average, 15.9 emails per sender; those who indicated they would be willing to send postcards estimated they would send, on average 14.3 postcards per sender.

### CRC Screening Email Messages: Expected Impact

Close to three-quarters of all participants thought that the self-edited message could have a positive impact; 71.5% (313/438) thought receiving the edited message would make their friends and family more likely to discuss CRC screening with a health care provider, and 73.1% (320/438) would be more likely to discuss screening if they themselves received such a message.

### CRC Screening Email Messages: Reasons for Not Sending

While many participants in our study expressed willingness to share cancer-screening messages via email or e-communication, there are also important lessons to be learned from the 58/438 (13.2% of all participants) who were unwilling to send messages. Asked about their reasons for not sending this message, 43.1% (25/58) of those unwilling said they felt emails were inappropriate, 62.1% (36/58) expressed willingness to discuss the issue verbally. Additionally, 22.4% (13/58) cited their own limitations (lacked expertise) and 20.7% (12/58) felt their social network would not receive the message well (some felt their network members would be offended, while others said their network had already been screened). Equal percents (12.1%, 7/58) found the message unappealing and stated that they were already discussing this within their social network (and therefore didn’t need to send such a message). (Participants could provide more than one reason for not sending messages).

## Discussion

### Study Participants and Electronic Communication

When given both a template and an opportunity to create their own content, most study participants expressed willingness to pass along a personalized CRC screening message to members of their social network, and most thought the message would have a positive impact. Adults in this 40-70 year old age group were willing to share their cancer screening experience with peers and to promote screening using a variety of modes. Approximately one in ten had already used either email or electronic communication to discuss a cancer screening test. Most were regular email users and over one-third had discussed routine health topics via email. The majority had used another form of electronic communication such as text messaging or social media in the preceding week, with one in ten having used these modes for communication about routine health topics. Many adults expressed a willingness to use email and electronic communication to share cancer screening experiences.

Our findings are consistent with recent surveys [1,2,4] that reflect already high rates of email use and rising rates of social media use among adults in this age group. Data from the 2007 Health Information National Trends Survey (HINTS) showed that approximately one-quarter of Internet users had used social networking sites in the preceding year, but that relatively few older adults had done so (5.5% of those 65 years and over) [3]. By 2012, a Pew Internet poll showed 57% of Internet users 50-64 years old and 38% of those 65 years and older using social networking sites [1].

Our findings also align with recent studies demonstrating use of electronic communication to discuss health topics. Cycle 1 of HINTS 4, collected in 2011-2012, asked specifically about visiting a social networking site such as Facebook or LinkedIn “to read and share about medical topics” and found that 17.0% of Internet users had done this (12.9% of Internet users 50-64 years old, and 7.6% of Internet users 65-74 years old, unpublished data) [21].

Recent work indicates that Internet users may be receptive to the use of narratives to promote CRC screening within an online community [22]. While participation in Facebook support groups for breast cancer has been described among younger users [23], there is little documentation in the literature of older adults using Facebook or Twitter to discuss cancer or cancer screening. Social groups for prevention as well as support in CRC and breast cancer have been described in a recent content analysis [16], which identified 216 breast cancer groups and 171 CRC groups on Facebook and Twitter, but did not provide information on the age of participants.

Our study addresses the intersection of two distinct evolutions. The first is the spread of innovative and Internet-based technologies among older adults who are becoming increasingly comfortable both with text messaging and with social media platforms. The second is patients’ growing expectation that they will engage in collaborative and interactive dialogues around health.

### Adults Spreading the CRC Screening Message

As our next step, we plan to recruit insured patients 50-70 years old at the time of CRC screening completion, and invite them to spread messages promoting screening to network members via the pathway of their choice (eg, postcard, email, text messaging, and social media). We hypothesize that this approach would take advantage of new technologies [6], while remaining inclusive of motivated, but less technologically savvy adults. In addition to prompting Internet-based conversations, this approach might also encourage face-to-face or telephone discussions.

We found that adults with less education were just as willing to pass along a CRC screening message to friends and family members, but were more likely to favor postcards. Mean health literacy scores for those who would send messages via postcard were significantly lower than both email senders and those who chose not to send. Adults with less education and lower health literacy may have social networks with higher numbers of unscreened individuals; efforts to include this group in peer-recruiting interventions are therefore particularly important.

Exploring a participatory intervention with multiple choices for network communication might also allow for future adaptation as new technologies supersede those of today. Interventions should capitalize on increased connectivity among social network members, facilitating exchanges of support, and information around cancer screening. Caution must also be taken. At times, social network members may communicate unhelpful or even harmful information [24,25]; interventions encouraging user-generated health content must include provisions to address this issue.

### Potential Study Limitations

There are potential limitations to our study. Participants all had health insurance. Study participants may therefore not be representative of uninsured populations. Participants were asked to report whether they would be willing to forward messages to friends and family, but since they were not actually requested to send messages, it is possible that they overestimated their willingness to do so. Future studies are needed to assess whether these results are generalizable to the population at large, and whether people are in fact willing to forward personalized messages.

### Conclusions

In conclusion, the majority of adults 42-73 years old in our study were willing to promote cancer screening to peers, and many were willing to use email and e-communication to do so. As the use of Web 2.0 participative technologies continues to rise in this age group, email, text messaging, and social media may offer cost-effective ways to disseminate peer-to-peer cancer screening messages. Our study indicates, however, that interventions relying exclusively on newer technologies may miss adults with lower education and lower health literacy levels who would otherwise be willing to engage in peer-to-peer screening promotion. This is a critical moment for further research.

## Multimedia Appendix 1

Willingness to use email and social media for peer-to-peer cancer screening communication.

## Abbreviations

ANOVA: analysis of variance
CMLT-Listening: Cancer Message Literacy Test-Listening
CMLT-Reading: Cancer Message Literacy Test-Reading
CRC: colorectal cancer
CRN: Cancer Research Network
FCHP: Fallon Community Health Plan
HINTS: Health Information National Trends Survey
KPGA: Kaiser Permanente Georgia
KPHI: Kaiser Permanente Hawaii
PEPPI: Perceived Efficacy in Patient-Physician Interactions
