# Supplementary material for: Adult Willingness to Use Email and Social Media for Peer-to-Peer Cancer Screening Communication: Quantitative Interview Study
Source: JMIR Res Protoc. 2013 Nov 28;2(2):e52. doi: 10.2196/resprot.2886 (PMC3868965; doi:10.2196/resprot.2886)
Supplement: Supplementary file 1 [file resprot_v2i2e52_app1.pdf]

Willingness to Use Email & Social Media
for Peer-to-peer Cancer Screening Communication

Interview Guide

Note: The following questions were asked of study participants as part of a longer interview in the context of the main study session.

Media Usage Items:

How many days last week did you use a computer?
0 1 2 3 4 5 6 7

If "0" or "never": **Do you ever use a computer?**
Yes No

If "Yes": **Have you used a computer in the past 30 days?**
Yes No

If participant has indicated they do ever use a computer: **When you used a computer last week, did you use it to access the internet?**
Yes No

How many days last week did you use email?
0 1 2 3 4 5 6 7

If "0" or "never": **Do you ever use email?**
Yes No

If "Yes": **Have you used a email in the past 30 days?**
Yes No

Do you have a cell phone that you use to access the internet?
Yes No

D**o you use any other electronic communication approach (texting, facebook, instant messaging, online chatting, twitter, video chatting or any other?)**

Yes *No*

	If yes, Which of these do you use? [choose all that apply]	How many days last week did you use (0-7) [*ask for all that apply*]?
texting		
Facebook		
Instant messaging/online chatting		
Video chatting		
Twitter		
Other: _______________		

Social Network and Cancer Screening Items:

Great – thanks. Now I'm interested in your social network – that's all the people that you know and communicate with. Thinking of routine health topics – like cancer screening – we are interseted in the people that you communicate with about these topics. I'm going to give you a worksheet to keep in front of you for these next questions.

Give blank work sheet to participant along with pen

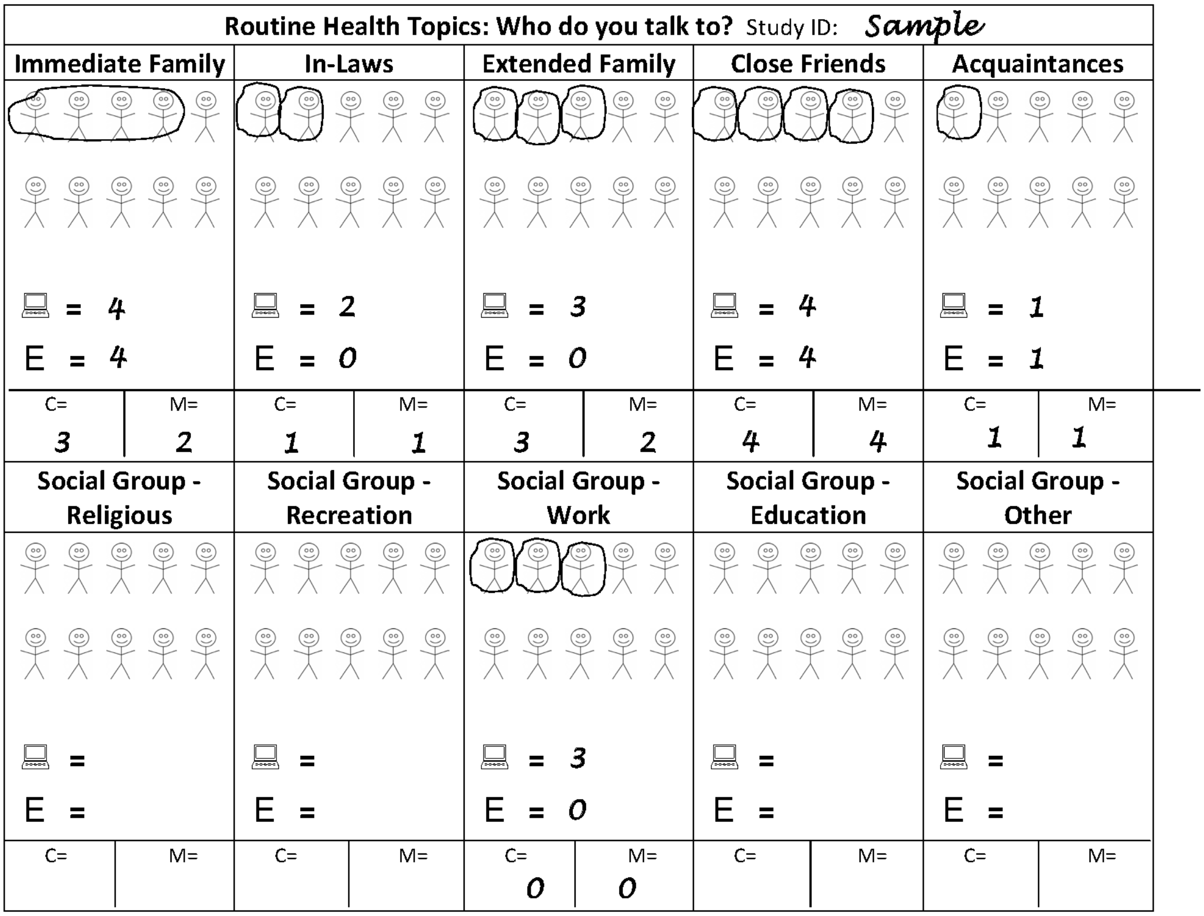

Routine Health Topics: Who do you talk to? Study ID: Sample				
Immediate Family	In-Laws	Extended Family	Close Friends	Acquaintances
💻 = 4<br>E = 4	💻 = 2<br>E = 0	💻 = 3<br>E = 0	💻 = 4<br>E = 4	💻 = 1<br>E = 1
C= 3	M= 2	C= 1	M= 1	C= 3	M= 2	C= 4	M= 4	C= 1	M= 1
Social Group - Religious	Social Group - Recreation	Social Group - Work	Social Group - Education	Social Group - Other
💻 =<br>E =	💻 =<br>E =	💻 = 3<br>E = 0	💻 =<br>E =	💻 =<br>E =
C=	M=	C=	M=	C= 0	M= 0	C=	M=	C=	M=

Please circle or somehow mark one stick figure for each person you can think of that you communicate with about routine health topics.

Here's a sample of what someone's work sheet could look like. *Show sample (see figure 2)*

- **We do plan to collect these at the end of our interview.**
- **But please don't worry about getting the categories perfect.**
- **Feel free to write down first names or initials in addition to circling – whatever you need in order to keep people in mind.**

Please think about *[indicate columns as you name them]*

- **members of your immediate family (parents, spouse or partner, children, siblings);**
- **in-laws;**
- **extended family (aunts, uncles, cousins, others);**
- **about close friends;   and more casual acquaintances;**
- **about people you know through religious or recreational activities, co-Workers, or others.**

Once again, these are people you would communicate with about routine health topics like cancer screening, vaccine shots, diet or exercise.

When participant appears to have completed the worksheet, wait one more moment to make sure you have given enough time.

When complete:
Thanks. Feel free to add people as we continue talking if you think of someone you've left off. I'll ask you a few follow-up questions now. From here on, I will refer to them as your family and friends.

If participant indicated they do not use email:
Based on your previous answers, I recall that you never use email. Do I remember correctly?

If yes: Then Skip to Other Electronic Communication
If no: Go to the next question below.

When discussing routine health topics with the people you've mentioned on your work sheet, do you ever use email?

Yes   No

If yes: **Have you ever used email to discuss colon cancer screening?**

Yes   No

If yes: **Have you ever used email to discuss mammography or breast cancer screening?**

Yes   No

If participant indicated they do not use other electronic communication methods:
Based on your previous answers, I recall that you do not use any other electronic communication approaches. Do I remember correctly?

If yes: skip to "Please imagine that…"
If no: proceed to next question.

When discussing routine health topics with the people you've mentioned on your work sheet, do you use any other electronic communication approach? (for example, texting, Facebook, online chatting, instant messaging, Twitter, videochatting or any other?)

Yes No

If yes: **what do you use?_**

Texting	*Facebook*	*Instant messaging/ online chatting*	*Video chatting*	*Twitter*	*Other:* ___________

Have you ever used another electronic communication approach to discuss colon cancer screening?

Yes No

Prompt: **For example, texting, facebook, online chatting, instant messaging, twitter, videochatting or any other?**

Have you ever used another electronic communication approach to discuss mammograms or breast cancer screening?

Yes No

P**lease imagine that:**

- **You completed colon cancer screening,**
- **Everything went OK and your results were fine, and**
- **The doctor asked you to help educate friends and family members over age 50 about colon cancer screening.**

How willing would you be to share your colon cancer screening experience with others…
. …In a conversation?

Very willing I might be willing Not at all willing

Omit if participant has answered that they do not use email at all.
….By email?

Very willing I might be willing Not at all willing

Omit if participant has answered that they do not use other electronic communication approaches at all.

…By another electronic communication approach?

Very willing   I might be willing   Not at all willing

For this next section, please continue to imagine that:

- **You completed colon cancer screening,**
- **Everything went OK and your results were fine, and**
- **The doctor asked you to help educate friends and family members over age 50 about colon cancer screening.**

We are trying to design a message to be sent out by people who have completed colonoscopies, so that they can explain to friends and family why screening is important.

Please help us design a message you'd be willing to pass along to friends and family members over age 50.

Give participant a copy of the message; do not give this page of the interview guide to the participant.

1. Please read the message all the way through.
2. Please cross out words or sentences that you feel uncomfortable with.
3. Please write in words or sentences that would make this sound more like a message you'd send to other people.

Take your time and when you finish, let me know – we have just a few more questions for you.

Hi, my doctor is asking me to pass this message along to people over age 50 who I know and care about, and I wanted to share it with you.

Colon cancer is expected to kill over 50,000 people in 2011.

Screening can stop colon cancer before it starts, or catch it early when it's likely to be easier to treat.

Everyone over age 50 should be screened.

There are a few ways to get screened for colon cancer. I just got a colonoscopy (which is one way to get screened).

If you want I'd be happy to talk to you about what my experience was like.

Have you had your screening done yet? If not, please consider talking to your doctor about screening options for colon cancer.

Now please take a look at the message with your changes.

If you were asked by a doctor or a health researcher <u>to help educate friends and family members over age 50 about colon cancer screening</u>, would you be willing to pass this message along either by email or postcard to any of the people listed on your work sheet?

Yes *No*

If no, clarify: **OK, that's good to know. Would you be willing to share your thoughts on why you wouldn't want to send out this message?**
If yes: **Which approach would you prefer: Email or Postcards that could be mailed or handed out? Or would you imagine sending a mix of these two?**

Email *Postcards* *Both*

If email: **What would be a good subject heading for this email? Can you think of what you could write that would make your family and friends likely to open up the email?**

Please use your work sheet to estimate how many people you'd send it to and tell us that number.

Email: ________________*people*

Mail or hand-delivered postcards: ________________*people*

[refer to the participant's edited message] **Do you think receiving this would make you more likely to discuss colon cancer screening with your healthcare provider?**

Yes *No* *Not Sure* *Already Been Screened, Wouldn't Change Anything*

If yes: **Why?**

If no: **Can you tell me why not?**

[refer to the participant's edited message] **Do you think this would make your friends and family -- that you send this message to -- more likely to discuss colon cancer screening with their healthcare provider?**

If yes: W**hy?**

If no: **Can you tell me why not?**

Thank you for your time today, this concludes our interview.
